# Economic Precariousness and the Transition to Parenthood: A Dynamic and Multidimensional Approach

**DOI:** 10.1007/s10680-022-09617-4

**Published:** 2022-04-20

**Authors:** Daniël C. van Wijk, Helga A. G. de Valk, Aart C. Liefbroer

**Affiliations:** 1 Netherlands Interdisciplinary Demographic Institute-KNAW/University of Groningen, The Hague, The Netherlands; 2 Department of Epidemiology, University Medical Centre Groningen, University of Groningen, Groningen, The Netherlands; 3 Department of Sociology, Vrije Universiteit Amsterdam, Amsterdam, The Netherlands

**Keywords:** Fertility, Precariousness, Income, Employment, Life course, Dynamic analysis

## Abstract

Economic precariousness has taken on a central role in explanations of the postponement of childbearing in developed societies. However, most studies conceptualize and operationalize precariousness as being static and one-dimensional, which provides only a partial perspective on the links between precariousness and fertility. In this paper, we study precariousness as a dynamic and multidimensional concept, distinguishing between past and current precariousness as well as between precariousness relating to income and to employment. Analyses are based on Dutch full-population register data. We select all inhabitants of the Netherlands who left education in 2006 and follow them until 2018. Event history analyses show that current and past income and employment precariousness all have independent negative effects on the first birth rate for men. Current and past employment precariousness and past income precariousness also reduce the first birth rate for women, but current income precariousness increases women’s probability of first conception. When precariousness is both persistent and multidimensional, it is associated with a threefold decrease in the monthly probability of conceiving a first child for men and almost a halving of the probability for women. Our analyses show the need for going beyond static and one-dimensional analyses in order to understand how economic precariousness may affect fertility behaviour.

**Supplementary Information:**

The online version contains supplementary material available at 10.1007/s10680-022-09617-4.

## Introduction

The notion of economic precariousness has taken on a central role in explanations of the postponement of childbearing in developed societies. As ongoing changes on the labour market have made the employment position of young adults increasingly precarious (Kalleberg, 2009; Standing, 2011), demographers and other social scientists have hypothesized that this has led them to delay major family formation transitions (e.g. Blossfeld et al., 2005). This was shown to be the case by aggregate (macro) level studies, which found that fertility levels decrease when economic conditions are weak (Schneider, 2015; Seltzer, 2019; Sobotka et al., 2011). It has been complemented by studies at the individual (micro) level, most of which have shown that the experience of economic precariousness delays childbearing (Adsera, 2011; Blossfeld et al., 2005; Hoffman et al., 2017; Laß, 2020; Wood & Neels, 2017). The vast majority of these studies has been rather static, focusing on the experience of precariousness at one point in time (mostly referring to the current situation). However, childbearing decisions are based on evaluations of one’s economic position over longer periods of time, and as such may be influenced not only by current precariousness but also by precariousness experienced in the past (Busetta et al., 2019; Ciganda, 2015). This is confirmed by recent studies that show that past experiences on the labour market affect fertility outcomes over and above the effect of the current economic position (Busetta et al., 2019; Ciganda, 2015; Schmitt, 2021). As a result, studies that take into account only the current situation will provide only a partial insight into the effect of precariousness on fertility.

In addition, most studies have been limited to one dimension of economic precariousness, measuring precariousness in terms of either (un)employment or (a low) income. One may, however, assume that income-related and employment-related precariousness have separate accumulating effects on fertility decisions, which makes it important to study these two dimensions together. This is in line with recent studies on labour market dynamics, which stress the dynamic and multidimensional nature of employment careers (e.g. Mattijssen & Pavlopoulos, 2019; Olsthoorn, 2014; Pohlig, 2019).

In this article, we move beyond previous studies on the effects of economic precariousness on fertility by conceptualizing and operationalizing precariousness as a dynamic and multidimensional concept, differentiating between past and current precariousness as well as between precariousness relating to income and to employment. We show that in order to gain a more complete overview of the impact of precariousness on fertility it is important to study how multiple forms of precariousness accumulate over time and among individuals. In addition, we examine how different combinations of current and past precariousness affect childbearing decisions. This allows us to explore how recent transitions into precariousness, recent transitions out of precariousness, and persistent precariousness affect fertility.

The focus of this study is on the Netherlands, a country that has recently witnessed a decline in fertility that is (among other factors) frequently attributed to the precarious economic situation of young adults (Stoeldraijer et al., 2019). Using Dutch administrative register data covering the entire population (Bakker et al., 2014), we link the income and employment histories of all men and women who left education in 2006 to information on the timing of the transition to parenthood up until 2018. The use of register data helps us to overcome some of the methodological issues of survey data, such as those associated with small sample sizes, selective nonresponse, recall bias (when using retrospective questioning), and sample attrition (in panel surveys). Register data also offer an advantage over survey data because of the availability of detailed income data. Moreover, register data are particularly useful for analysing past precariousness, as it has been shown that the complexity of work histories tends to be underestimated in survey data (Wahrendorf et al., 2019).

## Theoretical Background

Economic precariousness is a multidimensional concept, generally referring to a state of threatening insecurity or risk (Kalleberg, 2009; Olsthoorn, 2014; Vosko et al., 2009).^1^ In this study, we focus on objective indicators of precariousness. Following Olsthoorn (2014), we distinguish between precariousness relating to income on the one hand and precariousness relating to the employment situation on the other. Income precariousness pertains to the ability to secure a sufficient income, and is usually measured by a low total income derived from wages as well as other sources (Olsthoorn, 2014). Employment precariousness refers to an employment situation that is uncertain, unpredictable, and risky (Kalleberg, 2009). Joblessness perhaps constitutes the most obvious form of employment precariousness; in addition, workers in insecure employment relations such as temporary employment can be characterized as precarious (Kalleberg, 2009; Standing, 2011). Most previous research on fertility outcomes has focused on either income precariousness or employment precariousness. In the few studies that did include both types of precariousness, one of them was usually considered a control variable (Hart, 2015; Yu & Sun, 2018). Moreover, the majority of studies has focused on current precariousness. However, past precariousness may also have a considerable impact on childbearing decisions. Therefore, we place equal emphasis on both current and past precariousness in terms of income as well as employment. In what follows, we first discuss how current precariousness may influence the transition to parenthood and then consider how past precariousness could have an additional effect. We assess the impact of income and employment precariousness simultaneously. We then move on to discuss how different combinations of current and past precariousness may affect first birth rates. Finally, we comment on possible gender differences in the relationship between precariousness and fertility.

### Current Precariousness

Most recent studies expect that the experience of current economic precariousness makes both men and women postpone the transition to parenthood (e.g. Barbieri et al., 2015; Hart, 2015; Laß, 2020; Miettinen & Jalovaara, 2019; Wood & Neels, 2017). Three mechanisms have been argued to explain this relationship. First, precariousness increases financial strain and resource constraints (Brauner-Otto & Geist, 2018). As the experience of precariousness implies a lack of economic resources that are (perceived to be) necessary to raise a child, potential parents with limited resources may view children as too costly and therefore decide to postpone childbearing or to not have children at all (Auer & Danzer, 2015; Hart, 2015; Özcan et al., 2010). Second, precariousness has been argued to decrease fertility because it increases perceptions of economic uncertainty (Brauner-Otto & Geist, 2018). Precariousness will make people more uncertain about their ability to provide for their family in the future as well as about their future career path. This uncertainty may discourage them from making long-term binding commitments by becoming parents (Blossfeld et al., 2005; Chan & Tweedie, 2015). Moreover, strategic timing decisions may play a role, as having a child while being precariously employed may decrease the probability of finding stable employment in the future (Adsera, 2011; Laß, 2020). Third, the experience of economic precariousness increases stress and has detrimental effects on life satisfaction (Clark et al., 2001; Knabe & Rätzel, 2011), which may in turn inhibit childbearing. These three mechanisms all predict a direct effect of precariousness on fertility. At the same time, a lack of resources, increased uncertainty, and increased stress may also make individuals less attractive as a partner and may increase relationship conflict (Ishizuka, 2018; Smock et al., 2005). As a result, precariousness may also have an indirect effect on childbearing by decreasing union formation and union stability (Hart, 2015; Laß, 2020). Taken together, this leads us to hypothesize that *(H1) current income and employment precariousness decrease first birth rates*.

The results of recent studies largely support the hypothesis that current precariousness decreases first birth rates, although the evidence differs partially by gender, country, and the type of economic precariousness that is studied. Most prior research found lower first birth rates among men with lower incomes (Schmitt, 2012 in Germany but not the UK; Vignoli et al., 2012; Hart, 2015; Yu & Sun, 2018; Miettinen & Jalovaara, 2019; Van Wijk et al., 2021) and among men who are not employed (Lundström & Andersson, 2012; Pailhé & Solaz, 2012; Barbieri et al., 2015; Wood & Neels, 2017; Dupray & Pailhé, 2018; Miettinen & Jalovaara, 2019; but see Özcan et al., 2010; Schmitt, 2012; Begall, 2013; Raymo & Shibata, 2017; Yu & Sun, 2018; Laß, 2020). The evidence is more mixed for men’s temporary employment: several studies found that temporarily employed men postponed first births (Dupray & Pailhé, 2018; Lundström & Andersson, 2012; Pailhé & Solaz, 2012; Sutela, 2012; Vignoli et al., 2012), but most studies found no effect of men’s temporary employment (Auer & Danzer, 2015; Barbieri et al., 2015; Laß, 2020; Raymo & Shibata, 2017; Schmitt, 2012, 2021; Van Wijk et al., 2021; Vignoli et al., 2019). Turning to the evidence for women, the majority of past findings support the view that women with low incomes (Hart, 2015; Yu & Sun, 2018; Miettinen & Jalovaara, 2019; Van Wijk et al., 2021; but see Schmitt, 2012; Vignoli et al., 2012) and women in temporary employment (Lundström & Andersson, 2012; Pailhé & Solaz, 2012; Schmitt, 2012 in Germany but not the UK; Vignoli et al., 2012; Auer & Danzer, 2015; Barbieri et al., 2015; Dupray & Pailhé, 2018; Vignoli et al., 2019; Laß, 2020; Schmitt, 2021; Van Wijk et al., 2021; but see Raymo & Shibata, 2017) delay the transition to parenthood. The evidence for a delaying effect on first births of female joblessness is more mixed and seems to depend on the country that is studied, with negative associations being reported in Belgium (Wood & Neels, 2017), Sweden (Lundström & Andersson, 2012), and Finland (Miettinen & Jalovaara, 2019), but not in the Netherlands (Begall, 2013), Germany (Kreyenfeld, 2010; Özcan et al., 2010; Schmitt, 2012), the UK (Schmitt, 2012), Italy (Barbieri et al., 2015; Vignoli et al., 2012), and the US (Yu & Sun, 2018). In fact, in some countries jobless women are more likely to become mothers than employed women, which might be explained by the high opportunity costs of childbearing for employed women in societies where female employment and childrearing are (viewed as being) incompatible (Becker, 1981; Kreyenfeld, 2010).

### Past Precariousness

Most previous studies on the relationship between economic precariousness and fertility have focused on the current experience of precariousness, based on the assumption that the current experience will figure prominently in evaluations of the suitability of one’s economic position for raising a child and will have a direct impact on expectations about the future. This has been criticized by some recent studies, however, which argue that a focus on the current economic position provides an incomplete picture of the influence of precariousness on fertility behaviour (Busetta et al., 2019; Ciganda, 2015). These authors have argued that the decision to have a child is based on long-term evaluations of one’s economic situation, which are influenced more by the persistence of precariousness than by ‘snapshot indicators’ of the current situation (Busetta et al., 2019; Ciganda, 2015). This aligns well with the central proposition of life course studies that an individual’s prior life course influences later life outcomes (Huinink & Kohli, 2014; Mayer, 2009). Theoretically, it may be expected that the experience of past precariousness influences the transition to parenthood through similar mechanisms as current precariousness. First, past precariousness drains economic resources and may not have allowed people to build up a sound financial basis that can be used to invest in children (Kravdal, 2002). Second, the experience of past precariousness likely increases feelings of uncertainty and will decrease one’s confidence in having a stable and successful career in the future (Knabe & Rätzel, 2011). Those who experience long-term precariousness accumulate less human capital and face the threat of long-term future precariousness when having children (Adsera, 2004). Third, past precariousness has a negative, ‘scarring’ effect on life satisfaction even after taking into account the effect of the current position (Clark et al., 2001; Knabe & Rätzel, 2011), and lower life satisfaction in turn may inhibit childbearing. We therefore expect that *(H2) past income and employment precariousness decrease first birth rates over and above the effect of current precariousness*. Again, this may be a result of a direct effect of past precariousness on childbearing decisions as well as an indirect effect that runs through lower levels of union formation and union stability.

The empirical evidence that links past precariousness to the transition to parenthood is scarcer than that relating to current precariousness, and no studies were found that distinguished current and past income precariousness. Several studies have reported that past unemployment reduces first birth rates for men (Ciganda, 2015; Dupray & Pailhé, 2018; Pailhé & Solaz, 2012; Schmitt, 2021), whereas only one study found no such effect (Özcan et al., 2010). A similar negative effect was found for men’s past short-term employment in one study (Pailhé & Solaz, 2012), but men’s past temporary employment had no effect in another (Dupray & Pailhé, 2018). For women, past temporary or short-term employment reduced first birth rates in all studies that considered this indicator (Barbieri et al., 2015; Dupray & Pailhé, 2018; Pailhé & Solaz, 2012), whereas women’s past unemployment had no effect in some studies (Ciganda, 2015; Özcan et al., 2010; Pailhé & Solaz, 2012) but decreased first birth rates in others (Dupray & Pailhé, 2018; Schmitt, 2021).

### Precariousness Trajectories

As economic precariousness tends to cluster in time, some individuals will experience precariousness both in the past and in the present (Mattijssen & Pavlopoulos, 2019; Pohlig, 2019). On the one hand, we may expect that such long-term or persistent precariousness will have a particularly strong negative effect on first birth rates, as each additional experience of precariousness further decreases economic resources, increases perceptions of uncertainty about the future, and increases stress, which in turn causes a postponement of childbearing. As a result, we may expect that *(H3a) first birth rates are lowest among persons who experience persistent precariousness*. On the other hand, individuals who face persistent precariousness may realize that they are unlikely to ever fulfil the normative requirement of parenthood in the form of a stable job and income, and therefore disconnect their childbearing desires from economic conditions (Augustine et al., 2009). Moreover, those in a persistently precarious economic position may use parenthood as a way to provide meaning to their life (Edin & Kefalas, 2005) and to gain a source of security and social identity (Friedman et al., 1994) that they are unable to get from their employment career. In addition, as social benefits in the Netherlands increase when children are present in the household, having children may be a way for those in persistent precariousness to increase their income. As a consequence, first birth rates may increase when economic precariousness is persistent (Kravdal, 2002; Pailhé & Solaz, 2012). In contrast, when precariousness is experienced only in the past or only in the present, a re-evaluation of the requirements of parenthood becomes less likely as a stable employment career remains a feasible possibility, and childbearing may thus be postponed until the situation has improved. Therefore, a contrasting hypothesis to H3a is that *(H3b) first birth rates are higher among persons who experience persistent precariousness than among persons who experienced precariousness only in the past or only in the current situation*.

In addition, specific shifts in precariousness over time may matter for the decision to have a first child. On the one hand, persons who have recently made a transition out of precariousness, i.e. a transition from a precarious to a non-precarious state, may view their current situation as more favourable than persons who have continuously been in an advantageous position, and transitions out of precariousness may as such lead to heightened first birth rates. This may partly be a result of a ‘recuperation effect’ among individuals who postponed childbearing when they were in a precarious position in the past. We might thus expect that *(H4a) first birth rates are higher among persons who recently made a transition out of precariousness than among persons who did not experience precariousness at all.* Some evidence for the relevance of such transitions out of precariousness is provided by Barbieri et al. (2015), who found significantly higher first birth rates among Italian women who recently transitioned from an unstable to a permanent employment position. In contrast, Schmitt’s (2012) finding of a lower probability of having a first child among German and UK women who recently saw an increase in income and Begall’s (2013) finding of a lower first birth rate among women who recently made an upward career move contradict this expectation. On the other hand, it could be argued that a recent transition into precariousness, i.e. a transition from a non-precarious to a precarious situation, may increase first birth rates, as the advantageous situation in the past will have provided the necessary resources for having a child whereas the current precarious situation will decrease the opportunity costs of having children. Moreover, the stable situation in the past will make it more likely that the current state of precariousness is only temporary and may make one’s prospects of the future more positive. Finally, a selection effect may be at play here, as individuals who are planning to have a child may look for a more precarious situation that may be easier to combine with having children (Begall, 2013). A contrasting hypothesis to H4a is therefore that *(H4b) first birth rates are higher among persons who recently made a transition into precariousness than among persons who did not experience precariousness at all.* Schmitt’s (2012) finding that recent income losses increase first birth rates among German women supports this assumption, whereas he found the opposite effect for German men and no effects for men and women in the UK.

### Gender Differences

Our general expectation that economic precariousness inhibits childbearing among men and women alike is in line with recent studies (Brauner-Otto & Geist, 2018; Hart, 2015; Miettinen & Jalovaara, 2019). However, there are several reasons why this effect may be more ambiguous for women than for men. First, women in the Netherlands are still much more likely than men to decrease their working hours after becoming parents or to exit the labour market altogether (Statistics Netherlands & SCP 2018), and as a result opportunity costs will likely play a more prominent role in the childbearing decisions of women than they do for men. Second, societal norms may make parenthood an acceptable alternative to labour market participation for women but not for men, and may therefore make motherhood an attractive option (also) when faced with economic precariousness (Edin & Kefalas, 2005; Friedman et al., 1994). Therefore, the expectations that persistent precariousness and transitions into precariousness increase first birth rates might be more relevant for women than they are for men. On the other hand, strategic planning considerations may also be more relevant for women than for men, as women are more likely to temporarily leave the labour market when having a child and therefore may be more inclined to secure stable employment that allows them to return to work after childbearing (Laß, 2020). To take into account these gender differences, we analyse the impact of economic precariousness on the transition to parenthood separately for men and for women.

## Fertility and Labour Market Trends in the Netherlands

The Netherlands are a highly secular society where the use of birth control is widespread, concerns about population density are common, and there are no explicit pro-natalist policies (Fokkema et al., 2008; Mills, 2015). Further, there are strong norms that support childcare by the biological parents (usually the mother) and a reluctance of using full-time formal childcare, while a large share of mothers works part-time. The widespread availability of part-time employment has supported a ‘one-and-a-half-earner’ family model that can be argued to make the Netherlands occupy an alternative position that is in between the more traditional countries in Southern and Eastern Europe and the more gender egalitarian countries in Scandinavia (cf. Guetto et al., 2015). Also when it comes to fertility rates the Netherlands has remained at levels in between these two extremes in Europe. In recent years, however, fertility rates have declined, from a TFR of 1.796 in 2010 to 1.586 in 2018 (Statistics Netherlands, 2019). This drop in fertility largely resulted from a decrease in the number of births among women below age 30, and a corresponding rise in the age at childbearing (Stoeldraijer et al., 2019).

One of the explanations that is often given for this recent decline in fertility is the recent developments on the Dutch labour market (Stoeldraijer et al., 2019). First, the economic crisis that started in 2008 has had a long-term impact on the Dutch labour market, with an unemployment rate that peaked at 7.9% in February 2014 (Statistics Netherlands, 2020). Second, there has been a rapid shift towards flexible labour market arrangements, most prominently seen in the increase in employees with a temporary contract from 17.3% of all workers in 2008 to 22.5% in 2018 (De Vries & Chkalova 2020). In this study, we select a cohort of individuals who entered the labour market in 2006 and follow them until 2018, thus studying employment careers of individuals that had to establish themselves on the labour market during a turbulent period.

## Data and Methods

### Data

We use data from Statistics Netherlands’ System of Social Statistical Datasets (SSD), a system of interlinked registers containing information on the full population of the Netherlands. The SSD combines information from a wide range of administrative registers (e.g. tax and school registers), which are linkable by unique identifiers for individuals, households, buildings, and organizations (Bakker et al., 2014). The information we need for our study is available for the years 2006–2018. In order to obtain a good overview of the impact of current and past precariousness on the transition to parenthood, we need information on a person’s complete employment career since entering the labour market. In addition, individuals need to be followed for a relatively long time period in order to observe sufficient first births. To fulfil these two criteria, we select all individuals who left education in 2006 (i.e. an ‘education cohort’) and follow them until 2018. More specifically, we select all persons who (1) were living in the Netherlands on 1 January 2006; (2) were between 15 and 30 years old at that time; (3) were in education as their main activity^2^ somewhere in 2006; and (4) were not in education as their main activity anymore somewhere in 2007. We furthermore remove from the population everyone who returned to education later in the observation period (12.2% of the initial population) in order to exclude persons who left education only temporarily. Finally, we exclude everyone who had or conceived a child before leaving education (3.4% of the total population).

This selection leaves us with a population of 174,126 individuals that can be used in the analyses. Based on this population, we create a person-month file including both time-constant and time-varying covariates (Allison, 2014). The observation period starts in the month that a person left education and ends 9 months before the birth of a first child. Censoring takes place (a) 9 months before leaving the registers; or (b) in March 2018, whichever comes first. Observations are censored 9 months before a person left the registers—which may happen because of emigration or death—because we cannot be sure that a person did not conceive a child in these 9 months. Likewise, as data on births are available until December 2018, March 2018 is the final month for which we know whether someone conceived a child or not.

### Variables

Our dependent variable indicates whether a person conceived a first child during each monthly spell or not, calculated by ‘backdating’ the birth of the first child by 9 months. This variable is limited to live births [including adoptions, which constituted around 0.3% of all births in the Netherlands in the study period (Statistics Netherlands, 2014)].

*Current income precariousness* is defined by a person’s total income earned in the current month from employment, self-employment,^3^ and benefits. Incomes are measured before taxes are deducted, and are adjusted for inflation to January 2006 prices. We distinguish the following income categories: (1) below 1000 euros; (2) 1000–1499 euros; (3) 1500–1999 euros; (4) 2000–2499 euros (ref. cat.); (5) 2500–2999 euros; and (6) 3000 euros or more. *Past income precariousness* is measured using a categorical variable with the same categories and is based on the average monthly income between the start of the observation period and the current month, excluding the current month. The consequences of the decision to measure past income precariousness (as well as past employment precariousness, see below) over the entire observation period are evaluated in an additional analysis, in which the measurement of past precariousness is confined to months in the more recent past.

*Current employment precariousness* is measured by a person’s main activity in the current month. We distinguish between permanent employment (ref. cat.; this includes the self-employed who employ others), temporary employment (also including on call employees, temporary agency workers, and interns), self-employment, and different types of joblessness. Although based on the register data we are not able to distinguish between unemployment and inactivity as is often done (i.e. where persons are coded as unemployed only if they are actively searching for employment), we can differentiate different types of joblessness based on the type of benefits persons receive. A first category includes persons who receive unemployment benefits. Eligibility for receiving these benefits is based on previous employment experience. As a result, those who receive unemployment benefits have necessarily been employed in the recent past and can arguably be seen as the least precarious of all jobless persons. In contrast, social assistance benefits (bijstandsuitkering in Dutch) are not tied to previous employment, but are available to persons who can demonstrate that they are unable to make ends meet based on their own income and financial capital. We further distinguish a category of persons who receive illness, disability, or other benefits, referring to those who are unable to work due to disability or sickness. A last category comprises people who are jobless but do not receive any benefits. This includes a diverse group of people, ranging from persons who are looking for work but not eligible or willing to apply for benefits to persons who are voluntarily jobless.^4^*Past employment precariousness* is measured using a continuous variable for each of the main activity categories, which indicate the proportion of months that a person spent in that activity between the start of the observation period and the current month, excluding the current month. These variables range from 0 (no months in the past were spent in that activity) to 1 (all months in the past were spent in that activity). As stated above, additional analyses explore how the effect of past employment precariousness changes when its measurement is limited to the more recent past.

To measure precariousness trajectories, we create categorical variables that indicate whether persons were (1) not in a precarious situation in the past nor in the current month (stable non-precariousness; ref. cat.); (2) experienced precariousness only in the past (transition out of precariousness); (3) experienced precariousness only in the current month (transition into precariousness); or (4) experienced precariousness in the past as well as the current month (persistent precariousness). For *trajectories of income precariousness*, current precariousness is indicated by an income in the current month that is below 1500 euros (in January 2006 euros), whereas past precariousness is defined as having earned an average monthly income between the start of the observation period and the current month that was below 1500 euros. For *trajectories of employment precariousness*, the current situation is defined as precarious if a person is jobless, and the past is defined as precarious when a person was jobless for more than 20% of the months between the start of the observation period and the current month. We also use these definitions of current and past income and employment precariousness to create a variable that counts how many types of precariousness are experienced. This variable ranges from 0 (no precariousness at all) to 4 (precariousness is both persistent and multidimensional) and allows us to examine the total effect of the accumulation of precariousness on the first birth rate. All variables that measure precariousness (including those measuring the trajectories of precariousness and the accumulation of precariousness) are measured using time-varying variables, and as such their values change as persons’ income and employment careers unfold.

This combination of measures of economic precariousness provides a good overview of the experience of past and current income and employment precariousness by persons in our study population as measured by objective characteristics. At the same time, the register data do not contain subjective information on economic experiences, making it impossible for us to distinguish between the effects of decreased financial resources, increased uncertainty, and increased stress.

All models control for educational level, indicated by the highest level of completed education and measured on a yearly basis. We categorize this variable based on the International Standard Classification of Education (ISCED). The level of education is unknown for 4.1% of all person-months because it was not observed in the registers (e.g. because of education at a private institution or abroad). These person-months are assigned to a separate category termed ‘unknown’. The largest educational group (ISCED 3: higher secondary education) is chosen as the reference category. In addition, a time-constant variable is included that indicates a person’s ethnicity, based on his/her country of birth and the country of birth of his/her parents. Finally, age and duration since the start of the observation period are included as control variables, using both a linear and a quadratic term for age minus 15 as well as a linear and a quadratic term for the number of months since the start of the observation period. An interaction effect between educational attainment and age (including its squared term) is also included in all models to account for the strong variation in the age at first birth by educational attainment (Statistics Netherlands, 2012).

### Modelling Strategy

We use discrete-time event history analysis with logistic regression and standard errors clustered at the individual level to model how the monthly rate of conceiving a first child depends on the experience of economic precariousness. All models are estimated separately for men and women. Model 1 includes the variables capturing current income and employment precariousness as well as all control variables. Model 2 adds past income and employment precariousness. In Model 3, we substitute the variables measuring current and past precariousness for the variables that capture the trajectories of precariousness. Finally, Model 4 includes the number of types of precariousness to estimate the total effect of the accumulation of precariousness.

Because we use full-population register data with large numbers of observations, we do not report conventional tests of statistical significance in the paper. We do, however, include standard errors and Z-scores, which are useful when comparing the strength of the effects (Steenhof & Liefbroer, 2008). In addition, the results from the logit models are transformed to predicted probabilities calculated as estimated marginal means using Stata’s ‘margins’ command to illustrate the substantive significance of the results (Long & Mustillo, 2018; Williams, 2012). Given that absolute differences in monthly first birth probabilities are difficult to interpret, we focus on the relative differences in probabilities between precarious and non-precarious person-months.

### Descriptive Statistics

Table 1 shows the distribution of person-months across the dependent variable and the independent variables (*N* = 9,685,866 for men and *N* = 6,986,682 for women). On average, 0.47% of men and 0.76% of women in our data conceive a first child each month. At the end of the observation period, 49.77% of men and 66.84% of women have conceived a first child.

**Table 1:** Distribution of person-months across the dependent and independent variables

Categorical variables	Men, %	Women, %
Conception of a first child	0.47	0.76
*Current income*		
< 1000 euros	15.48	15.27
1000–1500 euros	14.74	17.65
1500–2000 euros	18.27	21.20
2000–2500 euros	18.31	19.11
2500–3000 euros	13.63	13.87
> 3000 euros	19.57	12.91
*Current employment position*		
Permanent employment	44.04	45.84
Temporary employment	36.39	38.84
Self-employment	5.31	3.13
Receiving unemployment benefits	1.50	1.31
Receiving social assistance benefits	1.94	1.15
Receiving illness, disability, or other benefits	4.90	5.08
Joblessness without income	5.92	4.65
*Past income*		
< 1000 euros	18.79	19.06
1000–1500 euros	21.03	23.19
1500–2000 euros	24.07	25.65
2000–2500 euros	17.57	18.79
2500–3000 euros	10.11	8.45
> 3000 euros	8.42	4.86
*Income trajectories*		
Stable non-precariousness	56.68	54.19
Transition out of precariousness	13.10	12.90
Transition into precariousness	3.49	3.56
Persistent precariousness	26.73	29.35
*Employment trajectories*		
Stable non-precariousness	76.93	80.56
Transition out of precariousness	8.81	7.26
Transition into precariousness	2.39	2.31
Persistent precariousness	11.87	9.88
*Number of types of precariousness*		
0	54.27	52.26
1	13.53	14.00
2	15.84	19.51
3	5.69	5.44
4	10.68	8.79
*Educational attainment*		
ISCED 0–1 [(pre-)primary education]	3.53	1.60
ISCED 2 (lower secondary education)	18.33	11.61
ISCED 3 (higher secondary education)	45.57	41.80
ISCED 4–6 (tertiary education, bachelor level)	18.27	26.07
ISCED 7–8 (tertiary education, master level)	9.75	15.38
Unknown	4.55	3.54
*Ethnicity*		
Native Dutch	80.17	81.59
Moroccan	2.63	2.13
Turkish	2.57	1.98
Surinamese	2.51	2.56
Antillean or Aruban	1.08	0.95
Other non-western	4.05	3.33
Other western	6.99	7.47

Continuous variables	Men, mean (SD)	Women, mean (SD)
Past temporary employment	0.45 (0.36)	0.50 (0.35)
Past self-employment	0.04 (0.15)	0.02 (0.11)
Past unemployment benefits	0.01 (0.04)	0.01 (0.03)
Past social assistance benefits	0.01 (0.08)	0.01 (0.07)
Past illness, disability, or other benefits	0.04 (0.17)	0.04 (0.17)
Past joblessness without income	0.08 (0.20)	0.07 (0.18)
Age	26.59 (4.40)	26.54 (4.17)
Months since start of observation	60.43 (38.60)	54.89 (37.43)

## Results

### Current and Past Precariousness

Results of the models including current (Model 1) and past (Model 2) income and employment precariousness are shown in Table 2 (the full regression models, which also include the effects of the control variables, can be found in the Supplementary Material). Model 1 shows that men who earn an income in the current month that is between 1000 and 1500 euros have the lowest likelihood of conceiving a first child, followed by men who earn less than 1000 euros. Above 1500 euros a month, men’s first birth rate increases more or less linearly with income. The results for the current employment position indicate that men who are self-employed have the highest likelihood to become fathers, followed by men in permanent employment. The first birth rate then decreases as employment states become more precarious. Temporarily employed men have a lower first birth rate than men in permanent employment, but a higher first birth rate than men who are jobless. Of all jobless men, men who receive unemployment benefits have the highest first birth rate, men who receive social assistance benefits or illness, disability, or other benefits have the lowest first birth rate, and men who are jobless but do not receive any benefits are somewhere in between these groups. The effects are quite substantial; for example, the probability to become a father for men who receive social assistance benefits is almost half that for men employed on a permanent contract (the average monthly probability to conceive a first child decreases from 0.50 to 0.25%). Overall, this shows that both current income precariousness and current employment precariousness strongly decrease first birth rates among men, which provides clear support for hypothesis H1.

**Table 2:** Logit coefficients, standard errors, and *Z*-scores of discrete-time event history models estimating the effect of current and past economic precariousness. Dependent variable: conception of first child^a^

	Men				Women			
	Model 1		Model 2		Model 1		Model 2	
	*b* (SE)	Z	*b* (SE)	*Z*	*b* (SE)	Z	*b* (SE)	*Z*
*Current income*								
< 1000 euros	− 0.424 (0.027)	− 15.69	− 0.209 (0.030)	− 6.87	0.155 (0.021)	7.51	0.374 (0.024)	15.60
1000–1500 euros	− 0.528 (0.024)	− 21.92	− 0.305 (0.026)	− 11.65	− 0.022 (0.017)	− 1.32	0.171 (0.019)	8.81
1500–2000 euros	− 0.202 (0.017)	− 12.05	− 0.096 (0.018)	− 5.38	0.020 (0.014)	1.42	0.114 (0.015)	7.66
2000–2500 euros (ref. cat.)								
2500–3000 euros	0.114 (0.016)	7.25	0.031 (0.017)	1.89	− 0.011 (0.015)	− 0.75	− 0.091 (0.016)	− 5.78
> 3000 euros	0.254 (0.015)	17.46	0.038 (0.018)	2.10	− 0.032 (0.015)	− 2.04	− 0.218 (0.020)	− 11.01
*Current employment position*								
Permanent employment (ref. cat.)								
Temporary employment	− 0.086 (0.011)	− 7.71	− 0.024 (0.014)	− 1.76	− 0.221 (0.010)	− 21.79	− 0.175 (0.012)	− 14.12
Self-employment	0.099 (0.019)	5.10	0.093 (0.029)	3.21	− 0.202 (0.025)	− 7.97	− 0.210 (0.036)	− 5.82
Receiving unemployment benefits	− 0.163 (0.043)	− 3.83	− 0.169 (0.044)	− 3.82	− 0.337 (0.038)	− 8.76	− 0.334 (0.040)	− 8.39
Receiving social assistance benefits	− 0.684 (0.055)	− 12.51	− 0.437 (0.068)	− 6.41	− 0.600 (0.052)	− 11.45	− 0.476 (0.067)	− 7.12
Receiving illness, disability, or other benefits	− 0.708 (0.040)	− 17.80	− 0.125 (0.058)	− 2.17	− 0.727 (0.030)	− 24.59	− 0.173 (0.044)	− 3.91
Joblessness without income	− 0.424 (0.040)	− 10.47	− 0.279 (0.045)	− 6.26	− 0.390 (0.032)	− 12.25	− 0.201 (0.036)	− 5.65
*Past income*								
< 1000 euros			− 0.390 (0.033)	− 11.90			− 0.375 (0.027)	− 13.95
1000–1500 euros			− 0.367 (0.022)	− 16.56			− 0.323 (0.019)	− 16.75
1500–2000 euros			− 0.170 (0.016)	− 10.86			− 0.161 (0.015)	− 10.92
2000–2500 euros (ref. cat.)								
2500–3000 euros			0.088 (0.017)	5.19			0.119 (0.018)	6.81
> 3000 euros			0.261 (0.019)	13.77			0.227 (0.023)	9.88
Past temporary employment			− 0.045 (0.019)	− 2.37			− 0.031 (0.018)	− 1.78
Past self-employment			0.060 (0.044)	1.38			0.097 (0.056)	1.73
Past unemployment benefits			− 0.551 (0.183)	− 3.01			− 0.771 (0.188)	− 4.11
Past social assistance benefits			− 0.406 (0.120)	− 3.39			− 0.039 (0.106)	− 0.37
Past illness, disability, or other benefits			− 0.983 (0.083)	− 11.82			− 0.905 (0.063)	− 14.27
Past joblessness without income			− 0.355 (0.059)	− 5.97			− 0.486 (0.051)	− 9.46
Log pseudolikelihood	− 276,065.96		− 275,545.97		− 301,768.73		− 301,261.03	
AIC	552,205.9		551,187.9		603,611.5		602,618.1	
*N* person-months	9,685,866		9,685,866		6,986,682		6,986,682	

^a^ Controlled for the time since the start of the observation period (including a quadratic term), ethnicity, age (including a quadratic term), educational level, and an interaction between educational level and age (again including a quadratic term for age). See Table S1 in the Supplementary Material for the full models

Adding past precariousness in Model 2 shows that having earned an average monthly income in the past that was below 1500 euros lowers men’s first birth rates, even after taking into account the current income. Again, men’s likelihood to become fathers increases roughly linearly with income when past income exceeds 1500 euros a month. For example, having earned an average monthly income in the past that was below 1000 euros decreases men’s probability to conceive a child by almost a third compared to men whose past income was between 2000 and 2500 euros (the average monthly probability decreases from 0.50 to 0.34%). In addition, the variables measuring past employment precariousness show that a larger proportion of months spent in temporary employment and joblessness in the past is associated with a reduced first birth rate, after taking into account the effects of the current position. Whereas past temporary employment only has a small negative effect on the first birth rate, past experiences of all four types of joblessness strongly decrease the likelihood to become a father. These results confirm the hypothesis (H2) that past precariousness decreases first birth rates over and above the effect of current precariousness. In fact, past precariousness has a negative effect on first birth rates that is roughly similar in size to the effect of current precariousness, and model fit strongly improves after adding past precariousness to the model. Moreover, even though the negative effects of men’s current precariousness decrease once past precariousness is included in Model 2, current precariousness continues to decrease the first birth rate in most cases, showing that men’s experiences of current and past precariousness have independent negative effect on the transition to fatherhood.

Turning to the results for women, women’s current employment precariousness is found to have a strong negative effect on the first birth rate that is similar to that for men. Women in permanent employment have the highest likelihood to become mothers, followed by women who are self-employed and women who are working on a temporary contract. First birth rates are lowest for jobless women, particularly for those who receive social assistance benefits or illness, disability, or other benefits. In contrast, women’s current income generally has only small effects on the first birth rate, and contrary to what was expected women who earn less than 1000 euros in the current month are more likely to become mothers than women with a higher income. In sum, for women the hypothesis (H1) that current precariousness decreases the first birth rate is confirmed for employment precariousness but not for income precariousness.

The results of Model 2 show that women’s past income precariousness does have a strong negative effect on the first birth rate. Further, the effect of women’s current income precariousness becomes more clearly positive in Model 2. Thus, having earned a low income in the past decreases women’s first birth rate, whereas after taking into account the effect of past income precariousness women who currently earn a low income have a higher rate of becoming mothers. Women who experienced more months in the past in which they received unemployment benefits, received illness, disability or other benefits, or were jobless but did not receive any benefits also have a decreased likelihood of becoming mothers. For example, women who were jobless but did not receive any benefits in half of all months in the past are about one fifth less likely to conceive a first child than women who did not experience joblessness without benefits in the past (the average monthly probability to conceive a child decreases from 0.77 to 0.61%). In contrast, past temporary employment and past social assistance benefits have no effect on women’s first birth rate. To conclude, although there are some exceptions, the results for women generally also confirm the hypothesis (H2) that the experience of past precariousness lowers the first birth rate even after taking into account the effects of current precariousness. This is further supported by the finding that model fit substantially improves after including past precariousness in the model.

### Precariousness Trajectories

**Fig. 1:**
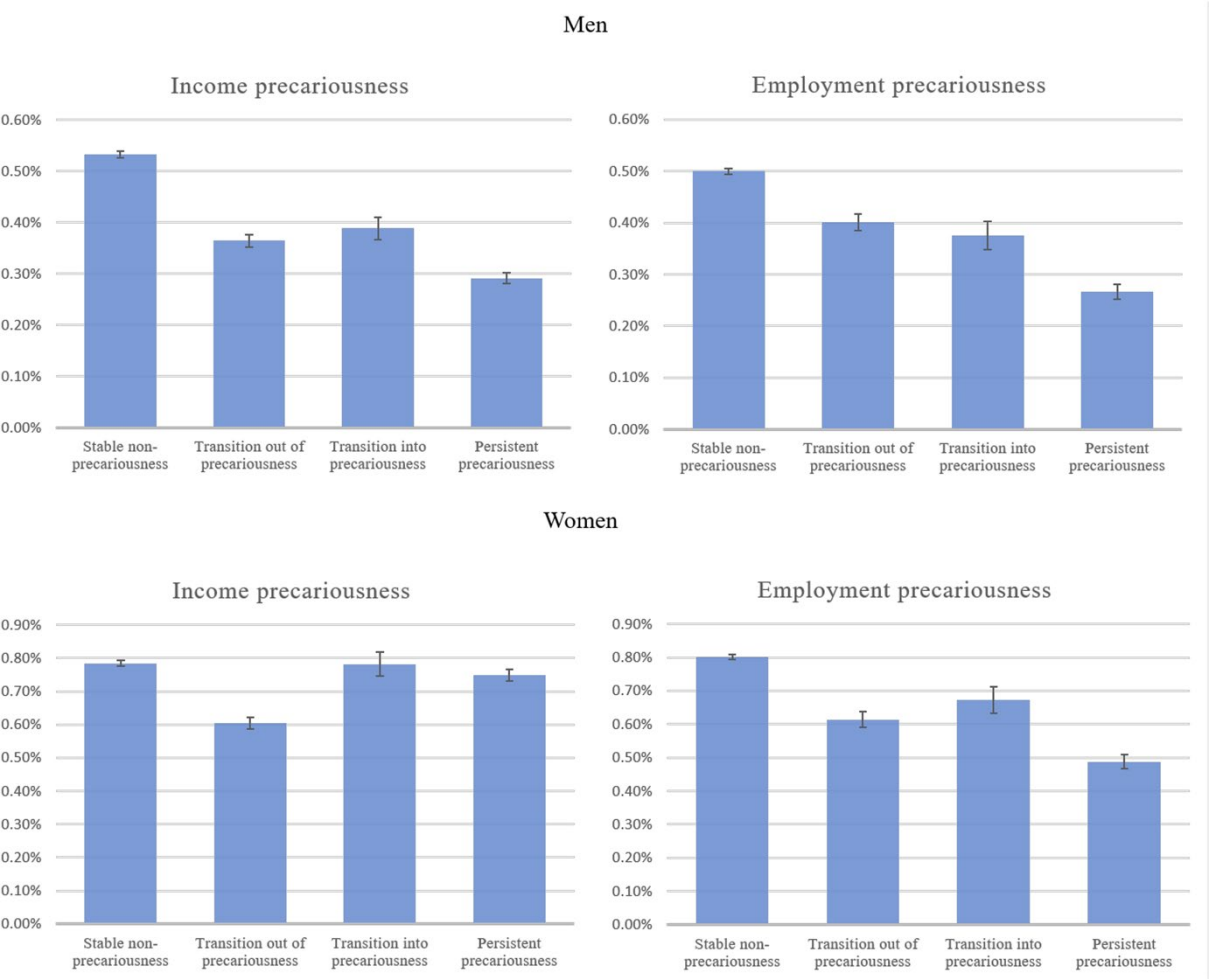
Predicted monthly probability of conceiving a first child by income and employment trajectories, based on Model 3

Model 3 (Table 3) shows how transitions into precariousness, transitions out of precariousness, and persistent precariousness influence the transition to parenthood. The results of this model are graphically illustrated in Fig. 1. This figure shows that men who experienced persistent income or employment precariousness have the lowest probability of conceiving a first child of all men, supporting hypothesis H3a but not H3b. Men who made a transition into precariousness or out of precariousness are more likely to conceive a first child than men who experienced precariousness in both past and present, but less likely than men who did not experience precariousness at all. In other words, each additional type of precariousness further decreases men’s probability of conceiving a first child, and this general pattern seems to prevail irrespective of whether a transition into or out of precariousness was made. This makes us reject hypotheses H4a and H4b for men. Model 4 (Table 4) shows that the total effect of the accumulation of precariousness is quite large: men who experience all four types of precariousness are more than three times less likely to have a first child than men who do not experience precariousness at all (the average probability of conception decreases from 0.58 to 0.17%).

**Table 3:** Logit coefficients, standard errors, and *Z*-scores of discrete-time event history models estimating the effect of precariousness trajectories. Dependent variable: conception of first child^a^

	Men		Women	
	Model 3		Model 3	
	*b* (SE)	*Z*	*b* (SE)	*Z*
*Income trajectories*				
Stable non-precariousness (ref. cat.)				
Transition out of precariousness	− 0.383 (0.019)	− 20.43	− 0.263 (0.016)	− 16.05
Transition into precariousness	− 0.319 (0.029)	− 10.84	− 0.003 (0.025)	− 0.12
Persistent precariousness	− 0.608 (0.021)	− 28.93	− 0.046 (0.015)	− 3.17
*Employment trajectories*				
Stable non-precariousness (ref. cat.)				
Transition out of precariousness	− 0.223 (0.021)	− 10.46	− 0.270 (0.021)	− 12.99
Transition into precariousness	− 0.287 (0.038)	− 7.59	− 0.177 (0.031)	− 5.72
Persistent precariousness	− 0.631 (0.029)	− 21.95	− 0.501 (0.023)	− 22.11
Log pseudolikelihood	− 276,159.73		− 301,826.84	
AIC	552,383.5		603,717.7	
*N* person-months	9,685,866		6,986,682	

^a^Controlled for the time since the start of the observation period (including a quadratic term), ethnicity, age (including a quadratic term), educational level, and an interaction between educational level and age (again including a quadratic term for age). See Table S2 in the Supplementary Material for the full models

**Table 4:** Logit coefficients, standard errors, and *Z*-scores of discrete-time event history models estimating the effect of the number of types of precariousness. Dependent variable: conception of first child^a^

	Men		Women	
	Model 4		Model 4	
	*b* (SE)	*Z*	*b* (SE)	*Z*
*Number of types of precariousness*				
0 (ref. cat.)				
1	− 0.401 (0.016)	− 24.29	− 0.211 (0.014)	− 14.79
2	− 0.611 (0.019)	− 31.86	− 0.154 (0.014)	− 11.18
3	− 0.828 (0.029)	− 28.55	− 0.299 (0.023)	− 13.01
4	− 1.228 (0.027)	− 46.07	− 0.552 (0.023)	− 24.18
Log pseudolikelihood	− 276,150.78		− 301,987.45	
AIC	552,361.6		604,034.9	
*N* person-months	9,685,866		6,986,682	

^a^Controlled for the time since the start of the observation period (including a quadratic term), ethnicity, age (including a quadratic term), educational level, and an interaction between educational level and age (again including a quadratic term for age). See Table S3 in the Supplementary Material for the full models

The effects of women’s employment trajectories reveal that women’s employment precariousness has a particularly strong negative effect on the first birth rate when it is persistent, i.e. when it is experienced in the present as well as the past, again supporting hypothesis H3a but not H3b (see Table 3 and Fig. 1). Women who did not experience employment precariousness in the past nor in the present (i.e. women who were stably employed) are most likely to become mothers, and women who made a transition into or out of precariousness are in between. Again, this provides little evidence for hypotheses H4a and H4b on the importance of transitions into and out of precariousness, but rather shows that each additional experience of employment precariousness further decreases women’s first birth rates. Results are different for women’s income precariousness, however, where it is found that first birth rates are lowest among women who made a transition out of precariousness (i.e. women who increased their income). Women who earned a stable high income and women who made a transition into precariousness are most likely to become mothers, and women with a persistent precarious income are only slightly below these two groups. This provides some support for the hypothesis (H4b) that making a transition into precariousness increases women’s first birth rates, although the first birth rate of women who made a transition into income precariousness does not differ from women who did not experience precariousness at all. Finally, results from Model 4 (Table 4) show that women’s first birth rate is particularly low when they experience all four types of precariousness, and the probability of having a first child nearly halves when women go from none to all four types of precariousness (the average probability of having a first child decreases from 0.84 to 0.48%). This shows that even though the impact of the accumulation of precariousness on the probability of conceiving a first child is smaller for women than it is for men, the effect of women’s precariousness is still quite substantial.

### Changing the Period Used to Measure Past Precariousness

In the above models, the measurement of past precariousness incorporates the income and employment histories of individuals across the entire observation period, from the start of the observation up until the current month. It may well be, however, that more recent experiences of precariousness have more pronounced effects on childbearing decisions than experiences of precariousness in the distant past. Therefore, we re-estimate Model 2 to explore how results change when we confine the measurement of past precariousness to more recent spells or when we assign a higher weight to more recent experiences (see Supplementary Material S2 for a more detailed explanation). The results of these additional models show that taking into account the entire observation period—as was done above—provides the best fit for the data, and model fit gradually decreases as the measurement of past precariousness is restricted to more recent periods. This implies that precariousness has long-lasting negative effects on the transition to parenthood, and it is of little importance whether a person was recently in a precarious position or whether precariousness was experienced in the more distant past.

## Discussion

Economic precariousness has often been suggested to explain postponement of childbearing in developed societies. As precariousness depletes financial resources, increases uncertainty about future employment and income, and engenders stress, it is often thought to cause delays in the transition to parenthood or even to make people put off childbearing altogether (Blossfeld et al., 2005; Miettinen & Jalovaara, 2019; Pailhé & Solaz, 2012; Vignoli et al., 2019). Most studies have, however, treated economic precariousness as a static and one-dimensional concept, focusing on the income or employment position at one point in time. In this study, we argue that in order to gain a more complete understanding of the impact of economic precariousness on fertility behaviour, multiple dimensions of precariousness should be included, both in the present and in the past. Our results support this view, as it was found that precariousness inhibited the transition to parenthood particularly when it accumulated over time and along multiple dimensions. When precariousness was both persistent and multidimensional, it was associated with a threefold decrease in the monthly probability of conceiving a first child for men and almost a halving of the probability for women. This is an effect that is much larger in magnitude than the effect found in most previous studies that focused on only one or two types of precariousness, and as such the total effect of economic precariousness on fertility behaviour may have been underestimated so far.

The findings of our study support recent calls for a more dynamic conceptualization of economic conditions when studying fertility behaviour, which takes into account not only the current situation but also prior experiences (Busetta et al., 2019; Ciganda, 2015). Past precariousness clearly impeded childbearing beyond the effect of the current situation, and the effect of past precariousness was similar to—and in some cases even larger than—the effect of precariousness experienced in the present. This fits well with theoretical arguments that individuals base the decision to become a parent on evaluations of their longer-term economic prospects, which are influenced not only by their current position but also by their past experiences.

We found no support for the expectation that first birth rates increase again when precariousness is persistent. In addition, in most cases we found little evidence that having made a transition into or out of precariousness had a substantial effect on the transition to parenthood. Rather, each additional experience of precariousness further reduced the first birth rate, independent of the experience of precariousness at other times. Moreover, additional analyses showed that the effects of past precariousness were not restricted to experiences in the recent past, but recent and more distant experiences of precariousness had similar consequences for the transition to parenthood. This also means that there is little evidence for a ‘recuperation’ of lost fertility taking place after having moved into a more advantageous position; instead, the experience of economic precariousness seems to have long-lasting consequences for fertility, both currently and in the future.

In addition, our study shows that income and employment precariousness have unique effects on the transition to parenthood. For income precariousness, men who earned less than 1500 euros a month had the lowest likelihood of becoming fathers while first birth rates increased more or less linearly with income after that point. The effect of women’s income precariousness was time-dependent: women who currently earned a low income were more likely to become mothers, whereas women who earned a low income in the past had a decreased probability to have a first child. Regarding employment precariousness, we found that first birth rates decreased as employment positions became more precarious for men and women alike. Distinguishing between different types of joblessness showed that men and women who received unemployment benefits were more likely to conceive a first child than men and women who received social assistance benefits or illness, disability, or other benefits, again supporting the view that the most precarious employment positions constitute the strongest impediments to childbearing. All in all, this calls for a much more nuanced and multidimensional study of the relationship between economic conditions and fertility in the future.

Whereas employment precariousness was associated with delays in the transition to parenthood in quite similar ways for men and women alike, the impact of income precariousness was much stronger and more consistent for men than it was for women. This may indicate that male breadwinner norms still figure prominently in the childbearing behaviour of Dutch men. Additionally, it might be explained by the higher opportunity costs of parenthood for Dutch women due to the (perceived) incompatibility of full-time employment and motherhood. This is illustrated by the finding that—after taking into account their past experience of precariousness—women who currently experienced income precariousness had a higher first birth rate than women who did not. Moreover, a particularly low probability of becoming a mother was found among women who had made a transition out of income precariousness, which may be due to a combination of high opportunity costs and a limited accumulation of economic resources in the past (see also Schmitt, 2012; Begall, 2013). Selection effects may also be at play here, as more family-oriented women might select into part-time employment, which often goes with a precarious income, even before conceiving a child. In contrast, transitions out of income precariousness could be more likely among more career-oriented women, who may postpone motherhood until a stable income position has been achieved. Future research should examine to what extent this pattern is unique for the Dutch context, with its high share of mothers who work part-time and the concomitant dominance of the ‘one-and-a-half-earner model’. On the one hand, this may have caused the effect of women’s current precariousness to be less consistent in the Netherlands than in countries where full-time maternal employment is more common (e.g. in Scandinavia; see Lundström & Andersson, 2012; Hart, 2015; Miettinen & Jalovaara, 2019). On the other hand, in societies—or among certain subgroups—where female employment and motherhood are (viewed as) incompatible, women’s precariousness may actually increase fertility (e.g. Kreyenfeld, 2010; Schmitt, 2012), and motherhood may provide an attractive alternative to precarious employment (Edin & Kefalas, 2005; Friedman et al., 1994).

In addition to the importance of the country context, our results should be interpreted in the light of the specific cohort that was studied, namely all individuals who left education in 2006. Although labour market conditions were relatively favourable when this cohort entered the labour market, they started to decline after 2008 as a result of the financial crisis and the increasing flexibility in the labour market, the consequences of which can be seen in the relatively high rates of joblessness and—particularly—temporary employment in our population. Further, the selection of a still relatively young cohort implies that we are studying fertility trends in very recent years. This—together with ongoing trends towards rising female educational attainment, increasing gender equality, and increasing maternal employment—might also be one of the factors that explain why in contrast to earlier studies in the Netherlands jobless women in our study population postpone the transition to parenthood (cf. Begall, 2013).

A few limitations of our study should be noted, and these provide useful starting points for future research. First, although we were able to follow individuals for a relatively long time period and as a result included the majority of first births in our analyses, our data did not cover all childbearing years. We did not include a small number of births that were conceived while persons were still in education. Furthermore, we observed individuals until up to thirteen years after they left education, and thus our results largely pertain to childbearing up until persons are in their mid-30s. This may also explain why we found little evidence for the recuperation of fertility after transitions out of precariousness, which might be more likely at older ages. Future work should therefore investigate whether the accumulation of precariousness has similar effects on the transition to parenthood at later ages, when biological constraints may make postponement a less attractive option. Second, we cannot draw conclusions about the direction of causality that produces the relationship between economic precariousness and the transition to parenthood. For example, part of the association may well be attributed to unobserved background characteristics that cause both economic precariousness and a low first birth rate (e.g. health; social capital). However, some recent studies that have utilized exogenous shocks that cause job losses to come closer to a causal interpretation of the effect of economic precariousness on fertility (Del Bono et al., 2015; Hofmann et al., 2017) suggest that the direction of the relationship is at least partly as hypothesized here. Third, we were unable to distinguish between the hypothesized mechanisms of earning an income that is perceived to be insufficient for family formation, perceptions of uncertainty about the future, and feelings of stress. An important way forward for future studies is to expand on our work by disentangling the mechanisms that link economic conditions and fertility, for example by measuring the perceived economic requirements for childbearing, expectations about future employment, and future family plans (see also Vignoli et al., 2020). Fourth, we have investigated the effects of precariousness at the individual rather than the couple level, and as such we did not take into account partnership dynamics nor answer the question how the distribution of precariousness within couples might influence childbearing decisions. We take this individual perspective as decreased union formation and union stability may be one of the ways in which precariousness might translate into lower birth rates. The data at hand are also best suited for a study focused on individuals. However, we acknowledge that taking a couple perspective could provide complementary insights that may help to further understand the impact of precariousness on fertility. In our study, this may be particularly relevant for part-time employed women, as the negative effects of these women’s lower earnings might be offset by the higher income of a potential partner. Finally, our focus on precariousness in terms of income and employment has ignored the potential consequences of precariousness in other domains, such as the family or the housing situation. Future studies could therefore benefit from a broader conceptualization of precariousness, taking into account not only the individual but also the partner, social network, and economic context.

To conclude, we found clear evidence for a negative relationship between economic precariousness and the transition to parenthood among Dutch men and women alike. It was especially the accumulation of precariousness, both in time and along multiple dimensions, that inhibited first childbearing. This shows that in order to gain a more comprehensive understanding of the influence of economic precariousness on fertility, precariousness should be treated as a dynamic and multidimensional concept.

## Supplementary Information

Below is the link to the electronic supplementary material.

Supplementary file1 (PDF 592 KB)

## Data Availability

The data that support the findings of this study are available from Statistics Netherlands but restrictions apply to the availability of these data, which were used under license for the current study, and so are not publicly available.

## Footnotes

1 We opted to use the term ‘precariousness’ rather than ‘uncertainty’. Even though the latter has frequently been used, we believe that ‘precariousness’ better captures the multidimensional nature of the concept under study. Moreover, by using the term ‘precariousness’ we do not assume beforehand that one particular mechanism (i.e. uncertainty) is dominant over others (e.g. low incomes, stress).

2 To measure educational enrolment and employment precariousness, we use Statistics Netherlands’ categorisation of states based on a person’s main activity in a given month. In most cases, a person’s main activity is the activity from which he or she earns the highest income. However, for persons enrolled in education, educational enrolment is seen as the main activity unless a person earns an income that is higher than the minimum monthly wage for those in full-time employment from any of the other activities.

3 Because data on the income of self-employees are only available on a yearly basis, it is assumed that this income was constant for the whole year.

4 We expect that the number of homemakers in our data is relatively small, as our population only includes young childless persons. In addition, some persons who are jobless but do not receive any benefits may be voluntarily jobless because they are taking time off before taking on employment responsibilities. This might make the designation ‘precarious’ less appropriate for a small part of this group.
