# Supplementary material for: Economic Precariousness and the Transition to Parenthood: A Dynamic and Multidimensional Approach
Source: Eur J Popul. 2022 Apr 20;38(3):457–83. doi: 10.1007/s10680-022-09617-4 (PMC9363546; doi:10.1007/s10680-022-09617-4)
Supplement: Supplementary file 1 — Supplementary file1 (PDF 592 KB) [file 10680_2022_9617_MOESM1_ESM.pdf]

Economic precariousness and the transition to parenthood: A dynamic and multidimensional approach

Supplementary Material

S1. Full regression tables

Table S1 Logit coefficients, standard errors, and Z-scores of discrete-time event history models estimating the effect of current and past economic precariousness. Dependent variable: conception of first child.

	Men				Women			
	**Model 1**		**Model 2**		**Model 1**		**Model 2**	
	b (SE)	Z	b (SE)	Z	b (SE)	Z	b (SE)	Z
Current income								
< 1000 euros	-0.424 (0.027)	-15.69	-0.209 (0.030)	-6.87	0.155 (0.021)	7.51	0.374 (0.024)	15.60
1000-1500 euros	-0.528 (0.024)	-21.92	-0.305 (0.026)	-11.65	-0.022 (0.017)	-1.32	0.171 (0.019)	8.81
1500-2000 euros	-0.202 (0.017)	-12.05	-0.096 (0.018)	-5.38	0.020 (0.014)	1.42	0.114 (0.015)	7.66
2000-2500 euros (ref. cat.)								
2500-3000 euros	0.114 (0.016)	7.25	0.031 (0.017)	1.89	-0.011 (0.015)	-0.75	-0.091 (0.016)	-5.78
> 3000 euros	0.254 (0.015)	17.46	0.038 (0.018)	2.10	-0.032 (0.015)	-2.04	-0.218 (0.020)	-11.01
Current employment position								
Permanent employment (ref. cat.)								
Temporary employment	-0.086 (0.011)	-7.71	-0.024 (0.014)	-1.76	-0.221 (0.010)	-21.79	-0.175 (0.012)	-14.12
Self-employment	0.099 (0.019)	5.10	0.093 (0.029)	3.21	-0.202 (0.025)	-7.97	-0.210 (0.036)	-5.82
Receiving unemployment benefits	-0.163 (0.043)	-3.83	-0.169 (0.044)	-3.82	-0.337 (0.038)	-8.76	-0.334 (0.040)	-8.39
Receiving social assistance benefits	-0.684 (0.055)	-12.51	-0.437 (0.068)	-6.41	-0.600 (0.052)	-11.45	-0.476 (0.067)	-7.12
Receiving illness, disability, or other benefits	-0.708 (0.040)	-17.80	-0.125 (0.058)	-2.17	-0.727 (0.030)	-24.59	-0.173 (0.044)	-3.91
Joblessness without income	-0.424 (0.040)	-10.47	-0.279 (0.045)	-6.26	-0.390 (0.032)	-12.25	-0.201 (0.036)	-5.65
Past income								
< 1000 euros			-0.390 (0.033)	-11.90			-0.375 (0.027)	-13.95
1000-1500 euros			-0.367 (0.022)	-16.56			-0.323 (0.019)	-16.75
1500-2000 euros			-0.170 (0.016)	-10.86			-0.161 (0.015)	-10.92
2000-2500 euros (ref. cat.)								
2500-3000 euros			0.088 (0.017)	5.19			0.119 (0.018)	6.81
> 3000 euros			0.261 (0.019)	13.77			0.227 (0.023)	9.88

Past temporary employment			-0.045 (0.019)	-2.37			-0.031 (0.018)	-1.78
Past self-employment			0.060 (0.044)	1.38			0.097 (0.056)	1.73
Past unemployment benefits			-0.551 (0.183)	-3.01			-0.771 (0.188)	-4.11
Past social assistance benefits			-0.406 (0.120)	-3.39			-0.039 (0.106)	-0.37
Past illness, disability, or other benefits			-0.983 (0.083)	-11.82			-0.905 (0.063)	-14.27
Past joblessness without income			-0.355 (0.059)	-5.97			-0.486 (0.051)	-9.46
Age	0.477 (0.014)	33.76	0.465 (0.014)	32.88	0.392 (0.014)	28.48	0.385 (0.014)	27.85
Age^2	-0.015 (0.001)	-29.53	-0.015 (0.001)	-29.05	-0.014 (0.001)	-26.69	-0.014 (0.001)	-26.46
Educational attainment								
ISCED 0-1 ((pre-)primary education)	2.976 (0.200)	14.91	2.939 (0.201)	14.64	3.076 (0.195)	15.79	3.118 (0.196)	15.90
ISCED 2 (lower secondary education)	1.426 (0.138)	10.30	1.382 (0.138)	10.01	1.961 (0.113)	17.38	1.931 (0.113)	17.07
ISCED 3 (higher secondary education; ref. cat.)								
ISCED 4-6 (tertiary education, bachelor level)	-2.169 (0.220)	-9.86	-2.125 (0.219)	-9.69	-2.701 (0.173)	-15.64	-2.674 (0.172)	-15.51
ISCED 7-8 (tertiary education, master level)	-4.323 (0.353)	-12.26	-4.124 (0.352)	-11.71	-6.646 (0.295)	-22.55	-6.418 (0.294)	-21.79
Unknown	1.950 (0.231)	8.46	2.027 (0.232)	8.76	1.826 (0.184)	9.90	1.960 (0.189)	10.35
*Educational attainment*age*								
ISCED 0-1*age	-0.391 (0.039)	-10.08	-0.361 (0.039)	-9.21	-0.432 (0.048)	-8.95	-0.413 (0.048)	-8.58
ISCED 2*age	-0.182 (0.023)	-7.79	-0.165 (0.023)	-7.09	-0.295 (0.021)	-14.04	-0.279 (0.021)	-13.18
ISCED 3*age (ref. cat.)								
ISCED 4-6*age	0.226 (0.029)	7.87	0.218 (0.029)	7.60	0.316 (0.025)	12.63	0.308 (0.025)	12.29
ISCED 7-8*age	0.447 (0.042)	10.55	0.421 (0.042)	9.93	0.735 (0.038)	19.45	0.702 (0.038)	18.59
Unknown*age	-0.250 (0.039)	-6.48	-0.242 (0.039)	-6.21	-0.270 (0.034)	-7.86	-0.268 (0.035)	-7.57
*Educational attainment*age^2*								
ISCED 0-1*age^2	0.012 (0.002)	6.55	0.011 (0.002)	5.81	0.012 (0.003)	4.38	0.012 (0.003)	4.19
ISCED 2*age^2	0.005 (0.001)	5.46	0.005 (0.001)	4.80	0.010 (0.001)	10.56	0.009 (0.001)	9.78
ISCED 3*age^2 (ref. cat.)								
ISCED 4-6*age^2	-0.005 (0.001)	-5.52	-0.005 (0.001)	-5.35	-0.008 (0.001)	-9.13	-0.008 (0.001)	-8.83
ISCED 7-8*age^2	-0.010 (0.001)	-8.19	-0.010 (0.001)	-7.70	-0.018 (0.001)	-15.18	-0.017 (0.001)	-14.45
Unknown*age^2	0.006 (0.002)	3.94	0.005 (0.002)	3.41	0.008 (0.002)	4.91	0.007 (0.002)	4.33

Months since start of observation	0.020 (0.001)	28.55	0.019 (0.001)	27.39	0.023 (0.001)	38.28	0.023 (0.001)	37.33
Months since start of observation^2	-0.000 (0.000)	-19.99	-0.000 (0.000)	-18.56	-0.000 (0.000)	-29.92	-0.000 (0.000)	-28.89
Ethnicity								
Native Dutch (ref. cat.)								
Moroccan	0.334 (0.030)	11.04	0.365 (0.031)	11.88	0.242 (0.032)	7.60	0.235 (0.032)	7.35
Turkish	0.472 (0.029)	16.43	0.494 (0.029)	16.95	0.266 (0.032)	8.18	0.270 (0.033)	8.26
Surinamese	0.133 (0.033)	4.08	0.160 (0.033)	4.86	-0.123 (0.031)	-3.93	-0.119 (0.031)	-3.80
Antillean or Aruban	0.260 (0.048)	5.47	0.275 (0.048)	5.73	-0.071 (0.050)	-1.41	-0.069 (0.050)	-1.39
Other non-western	0.032 (0.026)	1.22	0.062 (0.026)	2.36	-0.093 (0.027)	-3.44	-0.062 (0.027)	-2.29
Other western	-0.070 (0.019)	-3.61	-0.047 (0.020)	-2.41	-0.166 (0.018)	-9.16	-0.144 (0.018)	-8.01
Constant	-9.620 (0.091)	-105.67	-9.364 (0.093)	-100.99	-8.063 (0.079)	-102.19	-7.841 (0.081)	-97.33
Log pseudolikelihood	-276065.96		-275545.97		-301768.73		-301261.03	
AIC	552205.9		551187.9		603611.5		602618.1	
N person-months	9,685,866		9,685,866		6,986,682		6,986,682	

Table S2 Logit coefficients, standard errors, and Z-scores of discrete-time event history models estimating the effect of precariousness trajectories. Dependent variable: conception of first child.

	Men		Women	
	**Model 3**		**Model 3**	
	b (SE)	Z	b (SE)	Z
Income trajectories				
Stable non-precariousness (ref. cat.)				
Transition out of precariousness	-0.383 (0.019)	-20.43	-0.263 (0.016)	-16.05
Transition into precariousness	-0.319 (0.029)	-10.84	-0.003 (0.025)	-0.12
Persistent precariousness	-0.608 (0.021)	-28.93	-0.046 (0.015)	-3.17
Employment trajectories				
Stable non-precariousness (ref. cat.)				
Transition out of precariousness	-0.223 (0.021)	-10.46	-0.270 (0.021)	-12.99
Transition into precariousness	-0.287 (0.038)	-7.59	-0.177 (0.031)	-5.72
Persistent precariousness	-0.631 (0.029)	-21.95	-0.501 (0.023)	-22.11
Age	0.465 (0.014)	32.95	0.377 (0.014)	27.34
Age^2	-0.014 (0.001)	-28.63	-0.014 (0.001)	-25.97
Educational attainment				
ISCED 0-1 ((pre-)primary education)	3.144 (0.200)	15.70	3.300 (0.196)	16.87
ISCED 2 (lower secondary education)	1.490 (0.138)	10.78	2.002 (0.113)	17.77
ISCED 3 (higher secondary education; ref. cat.)				
ISCED 4-6 (tertiary education, bachelor level)	-2.336 (0.219)	-10.64	-2.768 (0.172)	-16.06
ISCED 7-8 (tertiary education, master level)	-4.361 (0.352)	-12.39	-6.613 (0.295)	-16.06
Unknown	2.180 (0.231)	9.45	1.991 (0.186)	10.73
*Educational attainment*age*				
ISCED 0-1*age	-0.396 (0.039)	-10.12	-0.468 (0.049)	-9.60
ISCED 2*age	-0.181 (0.023)	-7.78	-0.297 (0.021)	-14.11
ISCED 3*age (ref. cat.)				
ISCED 4-6*age	0.263 (0.029)	9.18	0.321 (0.025)	12.83
ISCED 7-8*age	0.474 (0.042)	11.20	0.724 (0.038)	19.18
Unknown*age	-0.278 (0.038)	-7.23	-0.298 (0.034)	-8.64
*Educational attainment*age^2*				
ISCED 0-1*age^2	0.012 (0.002)	6.30	0.014 (0.003)	4.77
ISCED 2*age^2	0.005 (0.001)	5.20	0.010 (0.001)	10.57
ISCED 3*age^2 (ref. cat.)				
ISCED 4-6*age^2	-0.006 (0.001)	-6.89	-0.008 (0.001)	-9.26
ISCED 7-8*age^2	-0.011 (0.001)	-8.98	-0.018 (0.001)	-14.92
Unknown*age^2	0.007 (0.002)	4.64	0.009 (0.002)	5.61
Months since start of observation	0.020 (0.001)	29.41	0.024 (0.001)	39.78
Months since start of observation^2	-0.001 (0.000)	-20.05	-0.000 (0.000)	-30.75
Ethnicity				
Native Dutch (ref. cat.)				
Moroccan	0.384 (0.030)	12.60	0.251 (0.032)	7.90
Turkish	0.515 (0.029)	17.79	0.280 (0.032)	8.63
Surinamese	0.161 (0.033)	4.91	-0.122 (0.031)	-3.91
Antillean or Aruban	0.270 (0.048)	5.64	-0.075 (0.050)	-1.51
Other non-western	0.063 (0.026)	2.38	-0.054 (0.027)	-2.02

Other western	-0.053 (0.019)	-2.73	-0.148 (0.018)	-8.25
Constant	-9.531 (0.091)	-104.74	-7.985 (0.079)	-101.29
Log pseudolikelihood	-276159.73		-301826.84	
AIC	552383.5		603717.7	
N person-months	9,685,866		6,986,682	

Table S3 Logit coefficients, standard errors, and Z-scores of discrete-time event history models estimating the effect of the number of types of precariousness. Dependent variable: conception of first child.

	Men		Women	
	**Model 4**		**Model 4**	
	b (SE)	Z	b (SE)	Z
Number of types of precariousness				
0 (ref. cat.)				
1	-0.401 (0.016)	-24.29	-0.211 (0.014)	-14.79
2	-0.611 (0.019)	-31.86	-0.154 (0.014)	-11.18
3	-0.828 (0.029)	-28.55	-0.299 (0.023)	-13.01
4	-1.228 (0.027)	-46.07	-0.552 (0.023)	-24.18
Age	0.465 (0.014)	32.98	0.369 (0.014)	26.86
Age^2	-0.014 (0.001)	-28.61	-0.014 (0.001)	-25.60
Educational attainment				
ISCED 0-1 ((pre-)primary education)	3.130 (0.200)	15.64	3.300 (0.194)	16.97
ISCED 2 (lower secondary education)	1.486 (0.138)	10.77	2.050 (0.113)	18.20
ISCED 3 (higher secondary education; ref. cat.)				
ISCED 4-6 (tertiary education, bachelor level)	-2.338 (0.219)	-10.66	-2.852 (0.173)	-16.52
ISCED 7-8 (tertiary education, master level)	-4.350 (0.352)	-12.36	-6.753 (0.295)	-22.88
Unknown	2.161 (0.231)	9.37	1.967 (0.185)	10.63
*Educational attainment*age*				
ISCED 0-1*age	-0.395 (0.039)	-10.09	-0.472 (0.048)	-9.80
ISCED 2*age	-0.180 (0.023)	-7.76	-0.305 (0.021)	-14.55
ISCED 3*age (ref. cat.)				
ISCED 4-6*age	0.264 (0.029)	9.19	0.331 (0.025)	13.25
ISCED 7-8*age	0.473 (0.042)	11.18	0.741 (0.038)	19.61
Unknown*age	-0.275 (0.038)	-7.18	-0.295 (0.034)	-8.60
*Educational attainment*age^2*				
ISCED 0-1*age^2	0.012 (0.002)	6.28	0.014 (0.003)	4.94
ISCED 2*age^2	0.005 (0.001)	5.18	0.010 (0.001)	10.98
ISCED 3*age^2 (ref. cat.)				
ISCED 4-6*age^2	-0.006 (0.001)	-6.90	-0.009 (0.001)	-9.67
ISCED 7-8*age^2	-0.011 (0.001	-8.96	-0.019 (0.001)	-15.36
Unknown*age^2	0.007 (0.001)	4.60	0.009 (0.002)	5.59
Months since start of observation	0.020 (0.001)	29.39	0.024 (0.001)	39.28
Months since start of observation^2	-0.000 (0.000)	-20.09	-0.000 (0.000)	-30.20
Ethnicity				
Native Dutch (ref. cat.)				
Moroccan	0.384 (0.030)	12.61	0.239 (0.032)	7.55
Turkish	0.517 (0.029)	17.87	0.268 (0.032)	8.26
Surinamese	0.161 (0.033)	4.93	-0.132 (0.031)	-4.24
Antillean or Aruban	0.270 (0.048)	5.62	-0.080 (0.049)	-1.62
Other non-western	0.064 (0.026)	2.44	-0.063 (0.027)	-2.34
Other western	-0.052 (0.019)	-2.67	-0.151 (0.018)	-8.46
Constant	-9.525 (0.091)	-105.02	-7.918 (0.079)	-100.68

Log pseudolikelihood	-276150.78	-301987.45
AIC	552361.6	604034.9
N person-months	9,685,866	6,986,682

S2. Changing the period used to measure past precariousness

To explore how the impact of past precariousness on first birth rates depends on the period over which past precariousness is measured, we re-estimate Model 2 (i.e. the model including current and past precariousness) but now measure past precariousness in (1) the entire observation period (the baseline model); (2) the past 48 months; (3) the past 36 months; (4) the past 24 months; (5) the past 12 months; and (6) the past 6 months. Finally, we estimate a model in which more recent months are given more weight in the measurement of past precariousness than months in the distant past. Specifically, the variables that measure past precariousness weighted by recency are calculated so that each additional month that has passed since a given month decreases the weight assigned to that month in the calculation of past precariousness by one percentage point. This means that the income or employment situation one month ago gains a weight of 99% in the calculation of past precariousness, the income or employment situation two months ago gains a weight of 98%, and so forth. To ensure that a complete observation period is available to measure past precariousness in all these specifications, all models are estimated for a subset of person-months that occurred more than 48 months after leaving education.

Results of these alternative measures of past precariousness are reported in Table S4. They support the main conclusions in the paper, namely that current and past precariousness have unique negative effects on first birth rates, except for women's current income precariousness which – after controlling for past income – increases the likelihood of conceiving a first child. Moreover, comparing the different models in Table S4 shows that the models in which past precariousness is measured over the complete observation period – i.e. the baseline models that were reported in the main

text – provide the best fit for the data. Model fit gradually decreases as the period used to measure past precariousness becomes shorter. Assigning a higher weight to more recent spells of past precariousness in the model in the last column of Table S4 also does not improve model fit. Theoretically, this indicates that the experience of economic precariousness has long-lasting negative effects on the first birth rate, and it seems to be of little importance whether the experience of precariousness happened in the distant past or whether it took place more recently. Looking at the coefficients, this seems to be mainly a result of a stronger effect of past joblessness and – to a lesser extent – past income when they are measured over a longer time period. The only effect that seems to become slightly stronger when past precariousness is measured in more recent periods is that of men’s past temporary employment, indicating that it is mainly recent spells of temporary employment that affect men’s transition to parenthood.

Table S4 Logit coefficients, standard errors, and Z-scores of discrete-time event history models estimating the effect of economic precariousness, with past precariousness measured in periods that differ in length. Dependent variable: conception of first child.[a]

**Men**														
**Past precariousness calculated for**	**Complete observation period**		**Past 48 months**		**Past 36 months**		**Past 24 months**		**Past 12 months**		**Past 6 months**		**Complete observation period, weighted by recency**	
	b (SE)	Z	b (SE)	Z	b (SE)	Z	b (SE)	Z	b (SE)	Z	b (SE)	Z	b (SE)	Z
Current income														
< 1000 euros	-0.194 (0.035)	-5.49	-0.135 (0.037)	-3.63	-0.122 (0.038)	-3.18	-0.103 (0.041)	-2.53	-0.107 (0.046)	-2.32	-0.176 (0.052)	-3.35	-0.131 (0.037)	-3.54
1000-1500 euros	-0.286 (0.031)	-9.29	-0.239 (0.032)	-7.44	-0.228 (0.033)	-6.85	-0.225 (0.035)	-6.40	-0.212 (0.039)	-5.49	-0.241 (0.043)	-5.56	-0.236 (0.032)	-7.37
1500-2000 euros	-0.094 (0.020)	-4.69	-0.064 (0.021)	-3.06	-0.057 (0.022)	-2.63	-0.057 (0.023)	-2.49	-0.067 (0.026)	-2.59	-0.063 (0.029)	-2.17	-0.062 (0.021)	-2.95
2000-2500 euros (ref. cat.)														
2500-3000 euros	0.039 (0.019)	2.13	0.024 (0.019)	1.24	0.025 (0.020)	1.28	0.023 (0.021)	1.11	0.024 (0.023)	1.03	0.019 (0.027)	0.70	0.026 (0.019)	1.38
> 3000 euros	0.052 (0.020)	2.58	0.045 (0.022)	2.19	0.051 (0.023)	2.24	0.056 (0.024)	2.30	0.054 (0.028)	1.96	0.076 (0.032)	2.35	0.029 (0.022)	1.33
Current employment position														
Permanent employment (ref. cat.)														
Temporary employment	-0.032 (0.015)	-2.10	-0.018 (0.017)	-1.07	-0.005 (0.018)	-0.27	-0.001 (0.021)	-0.07	0.005 (0.026)	0.18	0.003 (0.033)	0.10	-0.017 (0.017)	-1.03
Self-employment	0.092 (0.030)	3.00	0.102 (0.036)	2.83	0.119 (0.039)	3.02	0.112 (0.045)	2.50	0.122 (0.057)	2.14	0.178 (0.073)	2.45	0.093 (0.036)	2.60
Receiving unemployment benefits	-0.187 (0.049)	-3.85	-0.203 (0.049)	-4.12	-0.203 (0.050)	-4.08	-0.213 (0.051)	-4.18	-0.204 (0.055)	-3.71	-0.175 (0.062)	-2.83	-0.193 (0.050)	-3.89
Receiving social assistance benefits	-0.439 (0.077)	-5.72	-0.412 (0.084)	-4.92	-0.386 (0.088)	-4.41	-0.401 (0.093)	-4.30	-0.353 (0.109)	-3.23	-0.241 (0.128)	-1.89	-0.387 (0.084)	-4.62
Receiving illness, disability, or other benefits	-0.127 (0.063)	-2.00	-0.147 (0.075)	-1.97	-0.137 (0.080)	-1.72	-0.111 (0.087)	-1.27	-0.122 (0.104)	-1.18	-0.145 (0.127)	-1.14	-0.072 (0.072)	-1.00
Joblessness without income	-0.361 (0.055)	-6.61	-0.356 (0.058)	-6.15	-0.349 (0.060)	-5.83	-0.345 (0.063)	-5.48	-0.328 (0.071)	-4.62	-0.268 (0.080)	-3.37	-0.336 (0.057)	-5.85
Past income														
< 1000 euros	-0.359 (0.039)	-9.24	-0.390 (0.042)	-9.26	-0.388 (0.043)	-8.99	-0.400 (0.045)	-8.81	-0.353 (0.050)	-7.11	-0.246 (0.055)	-4.51	-0.417 (0.042)	-9.90
1000-1500 euros	-0.348 (0.025)	-13.71	-0.401 (0.029)	-13.65	-0.404 (0.031)	-12.99	-0.384 (0.034)	-11.44	-0.393 (0.038)	-10.36	-0.338 (0.043)	-7.92	-0.397 (0.029)	-13.87
1500-2000 euros	-0.154 (0.017)	-8.88	-0.171 (0.019)	-8.78	-0.178 (0.021)	-8.68	-0.169 (0.022)	-7.62	-0.142 (0.025)	-5.66	-0.148 (0.029)	-5.13	-0.194 (0.019)	-10.22
2000-2500 euros (ref. cat.)														

2500-3000 euros	0.072<br>(0.019)	3.83	0.096<br>(0.019)	5.08	0.088<br>(0.020)	4.52	0.096<br>(0.021)	4.64	0.096<br>(0.023)	4.13	0.102<br>(0.027)	3.75	0.076<br>(0.019)	4.00
> 3000 euros	0.258<br>(0.021)	12.47	0.230<br>(0.022)	10.51	0.223<br>(0.023)	9.72	0.219<br>(0.024)	8.98	0.222<br>(0.028)	7.96	0.193<br>(0.032)	5.95	0.254<br>(0.022)	11.58
Past temporary employment	-0.054<br>(0.022)	-2.52	-0.066<br>(0.020)	-3.24	-0.086<br>(0.021)	-4.09	-0.092<br>(0.023)	-4.08	-0.099<br>(0.027)	-3.65	-0.101<br>(0.034)	-2.97	-0.068<br>(0.022)	-3.08
Past self-employment	0.032<br>(0.048)	0.67	0.011<br>(0.045)	0.25	-0.018<br>(0.046)	-0.38	-0.013<br>(0.050)	-0.26	-0.031<br>(0.061)	-0.52	-0.098<br>(0.075)	-1.31	0.025<br>(0.047)	0.53
Past unemployment benefits	-0.946<br>(0.191)	-4.96	-0.485<br>(0.121)	-4.00	-0.340<br>(0.106)	-3.20	-0.174<br>(0.091)	-1.90	-0.086<br>(0.076)	-1.12	-0.046<br>(0.072)	-0.64	-0.632<br>(0.145)	-4.34
Past social assistance benefits	-0.695<br>(0.138)	-5.03	-0.502<br>(0.110)	-4.58	-0.510<br>(0.108)	-4.71	-0.443<br>(0.107)	-4.12	-0.475<br>(0.118)	-4.01	-0.602<br>(0.135)	-4.45	-0.600<br>(0.122)	-4.92
Past illness, disability, or other benefits	-1.068<br>(0.093)	-11.53	-0.787<br>(0.091)	-8.64	-0.754<br>(0.094)	-8.05	-0.746<br>(0.099)	-7.55	-0.667<br>(0.112)	-5.94	-0.622<br>(0.133)	-4.69	-0.969<br>(0.094)	-10.26
Past joblessness without income	-0.455<br>(0.075)	-6.03	-0.354<br>(0.077)	-4.59	-0.328<br>(0.078)	-4.23	-0.291<br>(0.078)	-3.69	-0.275<br>(0.083)	-3.31	-0.333<br>(0.088)	-3.81	-0.429<br>(0.081)	-5.29
Log pseudolikelihood	-215976.7		-216061.16		-216098.43		-216155.7		-216228.11		-216297.65		-216005.61	
AIC	432049.4		432218.3		432292.9		432407.4		432552.2		432691.3		432107.2	
N person-months	5,461,931		5,461,931		5,461,931		5,461,931		5,461,931		5,461,931		5,461,931	
**Women**														
**Past precariousness calculated for**	**Complete observation period**		**Past 48 months**		**Past 36 months**		**Past 24 months**		**Past 12 months**		**Past 6 months**		**Complete observation period, weighted by recency**	
	b (SE)	Z	b (SE)	Z	b (SE)	Z	b (SE)	Z	b (SE)	Z	b (SE)	Z	b (SE)	Z
Current income														
< 1000 euros	0.324<br>(0.031)	10.57	0.304<br>(0.032)	9.41	0.321<br>(0.034)	9.53	0.319<br>(0.036)	8.93	0.309<br>(0.040)	7.70	0.287<br>(0.045)	6.41	0.320<br>(0.032)	9.93
1000-1500 euros	0.160<br>(0.024)	6.70	0.139<br>(0.025)	5.49	0.146<br>(0.026)	5.54	0.150<br>(0.028)	5.33	0.142<br>(0.032)	4.44	0.106<br>(0.036)	2.93	0.154<br>(0.025)	6.12
1500-2000 euros	0.104<br>(0.018)	5.85	0.099<br>(0.019)	5.25	0.103<br>(0.020)	5.26	0.094<br>(0.021)	4.51	0.104<br>(0.024)	4.41	0.100<br>(0.026)	3.79	0.101<br>(0.019)	5.48
2000-2500 euros (ref. cat.)														
2500-3000 euros	-0.094<br>(0.018)	-5.12	-0.110<br>(0.019)	-5.77	-0.100<br>(0.020)	-5.04	-0.092<br>(0.021)	-4.44	-0.071<br>(0.023)	-3.04	-0.066<br>(0.027)	-2.44	-0.110<br>(0.019)	-5.84
> 3000 euros	-0.197<br>(0.022)	-8.84	-0.232<br>(0.024)	-9.60	-0.216<br>(0.025)	-8.47	-0.195<br>(0.027)	-7.20	-0.164<br>(0.031)	-5.37	-0.165<br>(0.035)	-4.73	-0.221<br>(0.024)	-9.26
Current employment position														
Permanent employment (ref. cat.)														
Temporary employment	-0.148<br>(0.015)	-10.12	-0.158<br>(0.016)	-10.05	-0.163<br>(0.017)	-9.77	-0.181<br>(0.019)	-9.77	-0.194<br>(0.023)	-8.48	-0.211<br>(0.028)	-7.41	-0.151<br>(0.016)	-9.54
Self-employment	-0.151<br>(0.040)	-3.77	-0.190<br>(0.046)	-4.15	-0.219<br>(0.049)	-4.45	-0.245<br>(0.055)	-4.46	-0.265<br>(0.068)	-3.92	-0.315<br>(0.083)	-3.77	-0.172<br>(0.046)	-3.76

Receiving unemployment benefits	-0.294<br>(0.045)	-6.59	-0.303<br>(0.045)	-6.74	-0.311<br>(0.046)	-6.83	-0.325<br>(0.047)	-6.99	-0.315<br>(0.050)	-6.35	-0.296<br>(0.056)	-5.30	-0.287<br>(0.045)	-6.32
Receiving social assistance benefits	-0.491<br>(0.084)	-5.83	-0.494<br>(0.093)	-5.34	-0.486<br>(0.098)	-4.97	-0.492<br>(0.105)	-4.68	-0.442<br>(0.121)	-4.67	-0.401<br>(0.135)	-2.98	-0.458<br>(0.093)	-4.90
Receiving illness, disability, or other benefits	-0.168<br>(0.053)	-3.18	-0.133<br>(0.059)	-2.25	-0.133<br>(0.063)	-2.11	-0.142<br>(0.070)	-2.04	-0.206<br>(0.085)	-2.43	-0.207<br>(0.102)	-2.04	-0.080<br>(0.058)	-1.37
Joblessness without income	-0.323<br>(0.052)	-6.25	-0.332<br>(0.054)	-6.11	-0.344<br>(0.056)	-6.12	-0.365<br>(0.059)	-6.15	-0.373<br>(0.066)	-5.69	-0.381<br>(0.073)	-5.22	-0.263<br>(0.054)	-4.91
Past income														
< 1000 euros	-0.328<br>(0.036)	-9.19	-0.251<br>(0.037)	-6.79	-0.280<br>(0.038)	-7.29	-0.266<br>(0.040)	-6.70	-0.250<br>(0.043)	-5.78	-0.226<br>(0.047)	-4.81	-0.263<br>(0.037)	-7.01
1000-1500 euros	-0.261<br>(0.024)	-11.04	-0.190<br>(0.025)	-7.50	-0.200<br>(0.027)	-7.52	-0.208<br>(0.028)	-7.32	-0.196<br>(0.032)	-6.10	-0.147<br>(0.036)	-4.10	-0.217<br>(0.025)	-8.62
1500-2000 euros	-0.132<br>(0.017)	-7.59	-0.096<br>(0.019)	-5.19	-0.100<br>(0.019)	-5.14	-0.078<br>(0.021)	-3.78	-0.095<br>(0.023)	-4.04	-0.089<br>(0.026)	-3.37	-0.106<br>(0.018)	-5.82
2000-2500 euros (ref. cat.)														
2500-3000 euros	0.121<br>(0.019)	6.19	0.144<br>(0.019)	7.56	0.105<br>(0.020)	5.36	0.090<br>(0.021)	4.31	0.050<br>(0.023)	2.15	0.043<br>(0.027)	1.61	0.156<br>(0.019)	8.12
> 3000 euros	0.232<br>(0.025)	9.15	0.282<br>(0.026)	10.94	0.249<br>(0.027)	9.33	0.215<br>(0.028)	7.68	0.164<br>(0.031)	5.29	0.160<br>(0.035)	4.54	0.255<br>(0.026)	9.83
Past temporary employment	-0.049<br>(0.022)	-2.28	-0.022<br>(0.019)	-1.14	-0.017<br>(0.019)	-0.88	-0.002<br>(0.021)	-0.09	-0.003<br>(0.024)	-0.14	0.006<br>(0.029)	0.19	-0.029<br>(0.021)	-1.35
Past self-employment	0.029<br>(0.066)	0.43	0.068<br>(0.060)	-1.14	0.100<br>(0.060)	1.67	0.117<br>(0.063)	1.85	0.115<br>(0.073)	1.58	0.156<br>(0.086)	1.81	0.051<br>(0.065)	0.78
Past unemployment benefits	-1.480<br>(0.211)	-7.02	-0.809<br>(0.132)	-6.13	-0.618<br>(0.113)	-5.49	-0.382<br>(0.093)	-4.10	-0.225<br>(0.075)	-3.00	-0.149<br>(0.070)	-2.14	-1.051<br>(0.158)	-6.68
Past social assistance benefits	-0.417<br>(0.146)	-2.85	-0.365<br>(0.123)	-2.98	-0.368<br>(0.122)	-3.02	-0.358<br>(0.123)	-2.90	-0.419<br>(0.133)	-3.15	-0.455<br>(0.143)	-3.19	-0.426<br>(0.138)	-3.09
Past illness, disability, or other benefits	-1.074<br>(0.077)	-13.98	-0.946<br>(0.074)	-12.81	-0.888<br>(0.076)	-11.76	-0.815<br>(0.080)	-10.25	-0.679<br>(0.091)	-7.43	-0.649<br>(0.106)	-6.12	-1.076<br>(0.077)	-13.99
Past joblessness without income	-1.118<br>(0.081)	-13.80	-0.861<br>(0.081)	-10.58	-0.739<br>(0.081)	-9.11	-0.596<br>(0.081)	-7.38	-0.461<br>(0.081)	-5.67	-0.376<br>(0.085)	-4.44	-1.129<br>(0.087)	-12.95
Log pseudolikelihood	-204477.39		-204609.06		-204669.23		-204744.07		-204833.47		-204875.36		-204546.42	
AIC	409050.8		409314.1		409434.5		409584.1		409762.9		409846.7		409188.8	
N person-months	3,512,611		3,512,611		3,512,611		3,512,611		3,512,611		3,512,611		3,512,611	

[a] Controlled for the time since the start of the observation period (including a quadratic term), ethnicity, age (including a quadratic term), educational level, and an interaction between educational level and age (again including a quadratic term for age).
